# Opportunistic coronary calcium progression on routine chest CT improves cardiovascular risk stratification in patients with inflammatory bowel disease: a multicenter study

**DOI:** 10.3389/fcvm.2026.1804400

**Published:** 2026-04-28

**Authors:** Qi Zhang, Yanxun Su, Chenyao Song, Ting Xu, Wenyan Liu, Jiaxin Cao, Binwei Guo, Lingjie Wang, Huajie Yue, Cheng Xu, Xuhui Zhao, Sijin Li

**Affiliations:** 1 Department of Radiology, First Hospital of Shanxi Medical University, Taiyuan, Shanxi, China; 2 Department of Nuclear Medicine, First Hospital of Shanxi Medical University, Taiyuan, Shanxi, China; 3 College of Medical Imaging, Shanxi Medical University, Taiyuan, Shanxi, China; 4 Department of Radiology, Shanxi Provincial People’s Hospital Affiliated to Shanxi Medical University, Taiyuan, Shanxi, China; 5 Department of Molecular Imaging Precision Medical Collaborative Innovation Center, Shanxi Medical University, Taiyuan, Shanxi, China

**Keywords:** atherosclerosis, cardiovascular disease, coronary artery calcium, inflammatory bowel disease, progression

## Abstract

**Background:**

Patients with inflammatory bowel disease (IBD) have increased atherosclerotic cardiovascular risk that may be underestimated by conventional factors. Whether coronary artery calcium (CAC) progression adds prognostic value beyond baseline CAC in IBD is unclear.

**Methods:**

In this multicenter retrospective cohort, 467 IBD patients without known atherosclerotic cardiovascular disease underwent ≥2 routine non-contrast chest CT scans (mean interval 21.2 months). CAC progression was defined as incident CAC (0 to >0), absolute progression (0 < baseline <100 with annualized increase ≥10), or relative progression (baseline ≥100 with annualized increase ≥10%). Major adverse cardiovascular events (MACE) were the primary outcome; incident atrial fibrillation (AF) was secondary. Cox proportional hazard regression was utilized to estimate hazard ratios (HRs) for time to MACE regarding CAC progression. Incremental value was assessed by C-index and continuous net reclassification improvement (NRI).

**Results:**

Over a median follow-up of 37 months, 59 patients had MACE and 41 developed AF. CAC progression occurred in 27.6% and predicted MACE (HR 7.41, *P* < 0.001), with graded risk (relative HR 10.31; absolute HR 8.14; incident HR 5.22; all *P* < 0.001). Adding CAC progression to conventional factors improved discrimination (C-index 0.67 vs. 0.73) and reclassification (NRI 0.22, *P* < 0.001), whereas baseline CAC added modest value (C-index 0.67 vs. 0.68; NRI 0.04, *P* = 0.021). CAC progression was also associated with incident AF.

**Conclusions:**

Opportunistic CAC progression assessment from routine chest CT improves cardiovascular risk stratification in IBD beyond conventional factors and baseline CAC, including among patients with zero baseline CAC.

## Graphical Abstract

**Figure:**
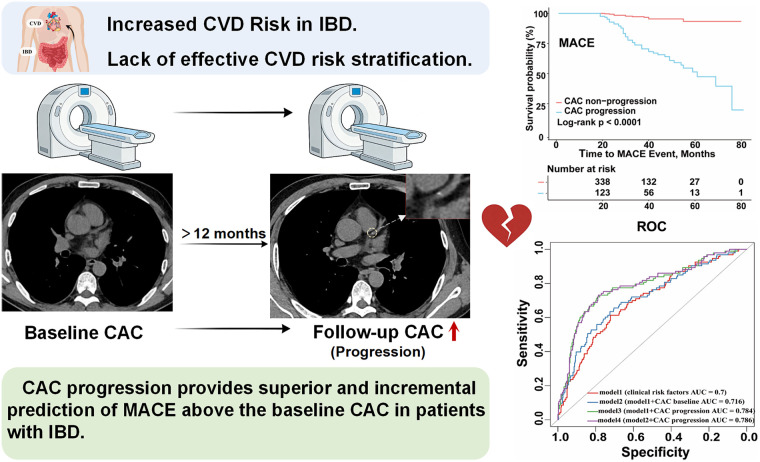

## 1 Introduction

Studies have increasingly demonstrated a link between inflammatory bowel disease (IBD) and cardiovascular diseases (CVD) (1, 2). Evidence has established that IBD patients present with higher rates of atherosclerotic cardiovascular disease (ASCVD) in spite of a low prevalence of the CVD risk factors (3). The underlying mechanism is likely driven predominantly by chronic systemic inflammation rather than traditional risk factors, which is consistent with the inflammatory hypothesis of atherosclerosis (4). The immune mediators characteristic of IBD, including macrophages, T cells (Th1 and Th17), interleukins (IL-6 and IL-12), tumour necrosis factor alpha (TNF*α*), and C-reactive protein (CRP), are similarly involved in the inflammatory pathways driving atherosclerotic plaque development (5). The European Society of Cardiology (ESC) guidelines recommend cardiovascular risk assessment should be considered in individuals with IBD (6). In contrast, contemporary American College of Cardiology/American Heart Association (ACC/AHA) prevention guidelines recognize chronic inflammatory conditions as risk-enhancing factors for ASCVD, but do not explicitly include IBD within formal risk stratification algorithms (7). Conventional risk calculators such as the ACC/AHA 10 year risk score (US) or SCORE2 (Europe) systematically underestimate risk in this population by failing to account for chronic inflammation. This gap underscores the urgent need for an appropriate tool for ASCVD risk assessment in IBD.

Coronary artery calcium (CAC) assessed by non-contrast computed tomography is a widely available marker for lifetime ASCVD risk in the general population (8, 9). The latest European and American prevention guidelines give CAC a class IIa recommendation for refining risk assessment in individuals with borderline or intermediate ASCVD risk (10, 11). Beyond a single CAC measurement, multiple cohort studies have reported that increases in CAC over time are linked to a 4- to 7-fold higher risk of ASCVD events, independent of baseline CAC levels and conventional risk factors (12–14). Nevertheless, clinical data evaluating the utility of CAC and its preventive implications in the IBD population remain scarce. Two studies have reported that baseline CAC scores are associated with subsequent major adverse cardiovascular events (MACE) in patients with IBD (15, 16). It is unclear, however, if the CAC progression over time also will refine cardiovascular risk stratification and offer incremental prognostic value in IBD.

Therefore, we assessed patients with IBD from two medical centers to elucidate the relationship between CAC progression and incident MACE, and to determine whether integration of CAC progression improves the predictive performance beyond conventional risk factors and baseline CAC scores. Furthermore, this study seeks to deepen the understanding of cardiovascular risk in IBD and to highlight IBD as a representative model for exploring the intricate interplay between intestinal pathology and cardiovascular disease, a concept increasingly recognized as the “gut-heart axis”.

## 2 Methods

### 2.1 Study population

This study is a retrospective analysis based on a prospective registry of complications in patients with IBD. We reviewed each patient's electronic medical records, imaging studies, colonoscopy reports, and corresponding pathology reports to ensure an accurate diagnosis of IBD, comprising ulcerative colitis (UC), Crohn's disease (CD), and indeterminate colitis. A total of 467 IBD patients without known ASCVD were enrolled from two tertiary hospitals in China. All participants underwent two or more non-contrast chest CT examinations with an interval of at least 12 months, from which CAC scores were derived. The first scan was performed between January 2018 and December 2022, and the last scan was completed before December 2023. In routine clinical practice at both institutions, chest CT in patients with IBD was commonly performed for IBD-related management or other non-cardiovascular indications, including disease follow-up, screening or evaluation of pulmonary complications, infection assessment, and preoperative evaluation. Importantly, chest CT examinations were not obtained for the purpose of CVD screening or risk assessment, but rather as part of standard care for IBD or for unrelated clinical indications. Patients with poor image quality, a history of statin use, incomplete clinical data, or insufficient follow-up information were excluded. In addition, patients who developed ASCVD before the follow-up CAC were also excluded. The study protocol complied with the Declaration of Helsinki and was approved by the Institutional Review Board (IRB) of the First Hospital of Shanxi Medical University (approval no. KYLL-2025-328) and the IRB of the Shanxi Provincial People's Hospital (approval no. KYLL-2025-951).

### 2.2 Coronary artery calcium score quantification

Scans were performed on a 64-slice multi-detector computed tomography (MDCT) scanner (GE Discovery, GE LightSpeed, SOMATOM Force, GE Revolution). Agatston CACS at both baseline and follow-up were calculated on non-contrast images from each chest CT scan using a dedicated cardiovascular post-processing workstation provided by United Imaging Healthcare (Shanghai, China). CAC was scored using the Agatston method, which was calculated for each calcified lesion, and the scores were summed across all lesions within a given artery and across all arteries (left main, left anterior descending, circumflex, and right coronary artery) to obtain the total calcium score. To ensure the validity of using opportunistic chest CT scans for CAC scoring, prior studies have shown excellent agreement between Agatston scores measured on non-gated chest CT and those obtained from dedicated ECG-gated cardiac CT scanning, including strong correlations and very good agreement across standard risk categories (17). Although non-gated imaging may slightly underestimate absolute scores, total scores and categorical severity are reliable for longitudinal assessment of progression. CAC was classified as none (0), mild (1–99), moderate (100–399) and severe (≥400). CAC progression was defined as one of the following groups: incident CAC, defined as baseline CAC = 0 with CAC > 0 at follow-up; absolute CAC progression, defined as 0 < baseline CAC < 100 with an annualized absolute CAC increase ≥10; and relative CAC progression, defined as baseline CAC ≥ 100 with an annualized percentage increase ≥10% (Formulas are presented in Supplementary Materials) (18, 19).

### 2.3 Outcome definitions and event ascertainment

The primary outcome was the occurrence of MACE, defined as a composite of myocardial infarction, hospitalization for unstable angina, ischemic stroke, transient ischemic attack, peripheral artery ischemia, revascularization, (percutaneous coronary intervention or coronary artery bypass grafting) and all-cause mortality. Myocardial infarction and unstable angina were defined according to contemporary guideline-based clinical diagnoses documented in the medical record (20). Ischemic stroke and transient ischemic attack were confirmed by neurological evaluation and imaging when available. Revascularization events included any coronary PCI or CABG performed during follow-up. Atrial fibrillation (AF) was recorded as a secondary outcome of interest, given its relatively high frequency in the IBD population. Incident AF was defined as the first clinically documented episode after the final CAC scan, confirmed by ECG, Holter monitoring, or cardiologist diagnosis. Patients were followed through clinic visits or telephone interviews. The final follow-up was conducted on December 31, 2024, which marked the end of follow-up. Patients were censored at the time of their last available follow-up or at the occurrence of the primary outcome. Follow-up time was defined as the time between the follow-up CT scan until a diagnosis of MACE, AF, loss to follow-up, or end of follow-up. The adjudication of all events was conducted by a seasoned radiology physician.

### 2.4 Statistical analysis

Categorical variables were reported as frequencies (percentages), whereas continuous variables were recorded as median (25th–75th quartiles) or mean ± standard deviation. Statistical significance for categorical variables was tested using the *χ*^2^ method and for continuous variables using the Mann–Whitney *U*-test or Student *t*-test. Because of its skewed distribution, annualized CAC progression was square-root transformed before assessing its associations with age and gender. The association between age and annualized CAC progression was examined using linear regression analysis, stratified by baseline CAC status (CAC = 0 vs. CAC > 0). Kaplan–Meier estimates were used to compute cumulative incidence of events by CAC progression, and the differences in estimates were compared using the log-rank procedure. Cox proportional hazard regression was utilized to estimate hazard ratios (HRs) with 95% confidence intervals (CIs) to MACE regarding CAC progression, with adjustment for covariates. To avoid model overfitting, covariates were selected based on clinical relevance and univariable associations with MACE (*P* < 0.01), and model parsimony was prioritized. Initially, Cox proportional hazard models were applied to adjust for risk factors (age, hypertension, diabetes). Additionally, Cox proportional hazard models were performed to further adjust for CAC progression groups (CAC incident, absolute CAC progression, relative CAC progression). The incremental value of the CAC baseline and CAC progression for predicting MACE were evaluated using the C-index for models including the baseline risk factors (Model 1), Model 1 + the baseline CAC (Model 2), Model 1 + CAC progression group (Model 3), and Model 2 + CAC progression group (Model 4). The continuous net reclassification index (NRI) was calculated to assess the reclassification ability of models at the median follow-up time. The Akaike information criterion (AIC) was used to measure the goodness of fit of models. Time-dependent receiver operating characteristic (ROC) curves, decision curve analysis (DCA), and calibration curves were used to evaluate the performance of Models 1–4 in predicting clinical outcomes. Statistical analyses were using R version 4.3.1 (Rstudio: Integrated Development for R) and SPSS version 27.0 (IBM Corporation). For all tests, a two-sided *P* value of <0.05 was considered statistically significant.

## 3 Results

### 3.1 Patient characteristics

**Figure 1:**
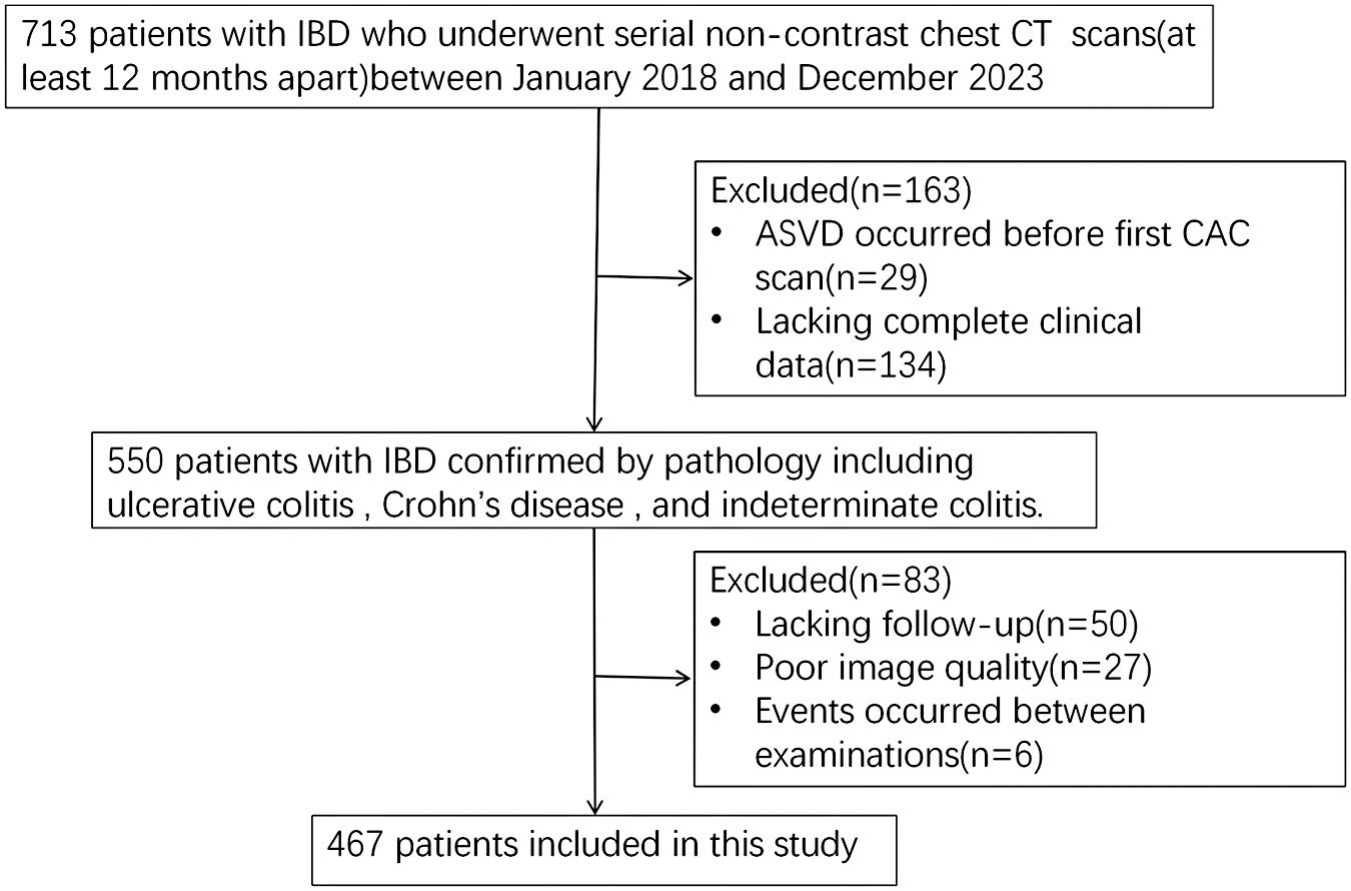
Flowchart of the study population. Flow diagram illustrating patient selection, inclusion, and exclusion criteria in the study cohort. IBD, inflammatory bowel disease; CAC, coronary artery calcium.

**Figure 2:**
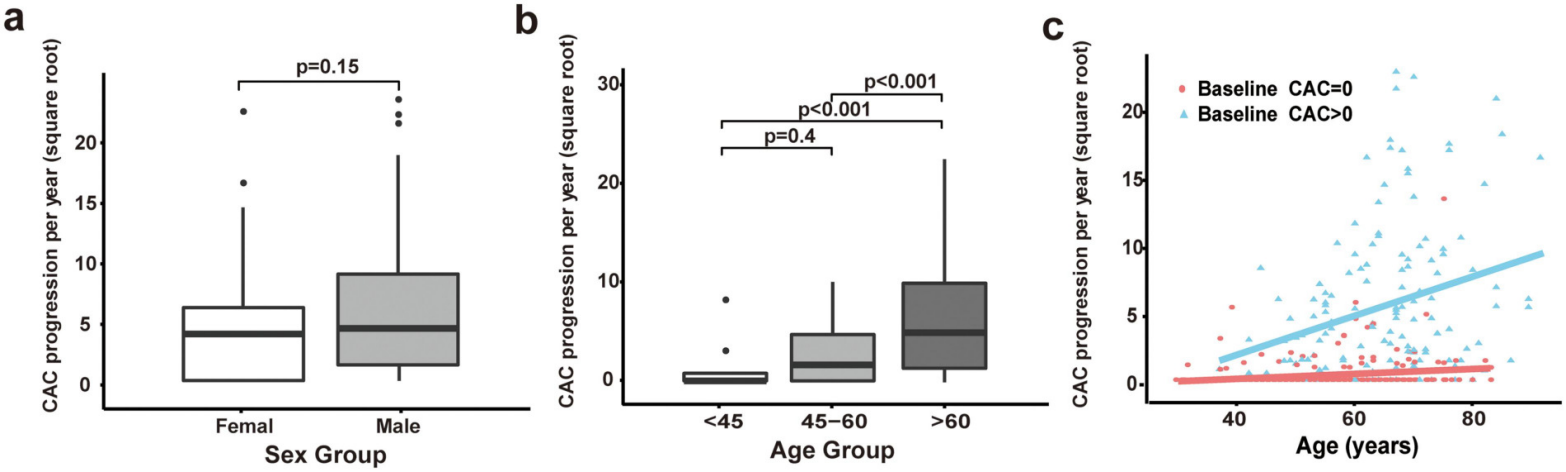
Distribution of annualized coronary artery calcium (CAC) progression by age and sex. **(a)** Annualized CAC progression stratified by sex. **(b)** Annualized CAC progression stratified by age groups (<45, 45–60, and >60 years). **(c)** Scatter plot showing the relationship between age and annualized CAC progression, stratified by baseline CAC status (CAC = 0 vs. CAC > 0).

A total of 467 IBD patients with two non-contrast chest CT examinations were included according to the inclusion and exclusion criteria (Figure 1). During a mean interscan period of 21.2 ± 9.7 months, 129 patients (27.6%) exhibited progression of CAC. Baseline characteristics of the study cohort stratified by the presence of CAC progression are presented in Table 1. The median age was 54 years (IQR: 45–64 years); 262 (56.1%) patients were male. With regard to traditional cardiovascular risk factors, the CAC progression group had a significantly higher prevalence of hypertension (39.5% vs. 17.5%, *P* < 0.001), diabetes (14.7% vs. 6.2%, *P* = 0.003), and a higher BMI (23.1 ± 3.3 kg/m^2^ vs. 22.3 ± 3.5 kg/m^2^, *P* = 0.033). No significant disparities in IBD-related characteristics (disease severity, disease type, disease duration, CRP) were observed between these two groups. The use of corticosteroids, biologics, and the level of TC and LDL also showed no significant differences. Supplementary Table S1 presents characteristics of study participants stratified by the baseline CAC. Patients with CAC progression were substantially more likely to have detectable CAC at baseline. A comparison between UC and CD was also performed (Supplementary Table S2), but no meaningful differences were observed. In addition, the distribution of CAC progression rates across gender and age categories is illustrated in Figure 2. CAC progression per year did not differ significantly between female and male patients (*P* = 0.15; Figure 2a). In contrast, CAC progression increased significantly with advancing age, with patients aged >60 years showing markedly higher progression rates compared with those aged 45–60 years and <45 years (both *P* < 0.001; Figure 2b). When analyzed continuously, age was positively associated with CAC progression. The association was modest but statistically significant among patients with baseline CAC = 0 (*R*^2^ = 0.04, *P* < 0.001) and stronger among those with detectable baseline CAC (*R*^2^ = 0.079, *P* < 0.001) (Figure 2c). Additional subgroup analyses are presented in Supplementary Tables S3, and S4. When stratified by baseline IBD disease activity, patients with active disease had higher CRP levels than those in remission (*P* = 0.002). In the analysis stratified by biologics use patterns, CAC progression did not differ significantly between groups (*P* = 0.297).

**Table 1:** Baseline characteristics of individuals stratified by progression of CAC.

Characteristic	Total	NONE CAC progression	CAC progression	*p* value
	(*n* = 467)	(*n* = 338)	(*n* = 129)	
Age, y	54 (45, 64)	50 (41, 60)	63 (55, 69)	**<0**.**001**
Male, %	262 (56.1)	176 (52.1)	86 (66.7)	**0**.**004**
Moderate to severe IBD, %	300 (64.2)	216 (63.9)	84 (65.1)	0.489
Disease type, %				0.087
UC	338 (72.4)	246 (72.8)	92 (71.3)	
CD	103 (22.1)	69 (20.4)	34 (26.4)	
IBD-U	26 (5.57)	23 (6.80)	3 (2.33)	
Diagnosis age, y	46 (35, 58)	43 (33, 55)	56 (45, 64)	**<0**.**001**
Disease duration, y	7.0 ± 7.6	6.7 ± 7.2	7.8 ± 8.6	0.149
Active disease at baseline, %	369 (79.0)	277 (82.0)	92 (71.3)	**0**.**012**
BMI, kg/m^2^	22.5 ± 3.4	22.3 ± 3.5	23.1 ± 3.3	**0**.**033**
Former smoker	63 (13.5)	37 (11.0)	26 (20.2)	**0**.**009**
Current smoker	50 (10.7)	34 (10.1)	16 (12.4)	0.464
Drinking, %	72 (15.4)	43 (12.7)	29 (22.5)	**0**.**009**
Hypertension, %	110 (23.6)	59 (17.5)	51 (39.5)	**<0**.**001**
Diabetes, %	40 (8.6)	21 (6.2)	19 (14.7)	**0**.**003**
Hyperlipidemia, %	132 (28.3)	89 (26.3)	43 (33.3)	0.133
TC, mmol/L	4.11 ± 1.09	4.15 ± 1.03	4.00 ± 1.23	0.230
LDL, mmol/L	2.08 ± 0.95	2.11 ± 0.91	1.99 ± 1.02	0.219
Corticosteroids, %	148 (31.7)	103 (30.5)	45 (34.9)	0.360
Aminosalicylates, %	407 (87.2)	288 (85.2)	119 (92.3)	**0**.**042**
Biologics at baseline, %	234 (50.1)	168 (49.7)	66 (51.2)	0.778
CRP, mg/L	16.8 ± 32.6	16.4 ± 33.5	17.9 ± 30.0	0.650
Baseline CAC=0, %	322 (68.95)	282 (83.43)	40 (31.01)	**<0**.**001**
Baseline CAC > 0, %	145 (31.05)	56 (16.57)	89 (68.99)	**<0**.**001**
Progression per year	0.0 (0.0, 2.7)	0.0 (0.0, 0.0)	23.2 (5.0, 76.8)	**<0**.**001**
CAC inter-scan time, m	21.2 ± 9.7	21.0 ± 8.8	21.7 ± 11.6	0.545

Significant *P*-values are printed in bold; CAC, coronary artery calcium; BMI, body mass index; SBP, systolic blood pressure; DBP, diastolic blood pressure; TC, total cholesterol; LDL-C, low-density lipoprotein-cholesterol; CRP, C-reactive protein.

### 3.2 Association of CAC progression with MACE

Over a median follow-up of 37 months (IQR: 29–47 months), 59 (12.6%) individuals experienced MACE and 41 (8.8%) AF. The individual components of MACE are detailed in Supplementary Table S5. Baseline characteristics of participants stratified by the MACE occurrence are summarized in Supplementary Table S6. Compared with the non-MACE group, patients who developed MACE were older (61.5 ± 11.2 vs. 53.0 ± 13.0 years, *P* < 0.001) and had higher proportions of drinking (27.1% vs. 13.7%, *P* = 0.008), hypertension (44.1% vs. 20.6%, *P* < 0.001), and diabetes (17.0% vs. 7.4%, *P* = 0.014). No significant differences were observed in sex distribution, BMI, smoking status, or lipid parameters including TC and LDL levels, although hyperlipidemia was more frequent in the MACE group (39.0% vs. 26.7%, *P* = 0.050). In the subgroup analysis by biologics use patterns, patients who used biologics at baseline or follow-up had a lower incidence of MACE than those without biologics use (Supplementary Table S4, *P* = 0.037).

**Figure 3:**
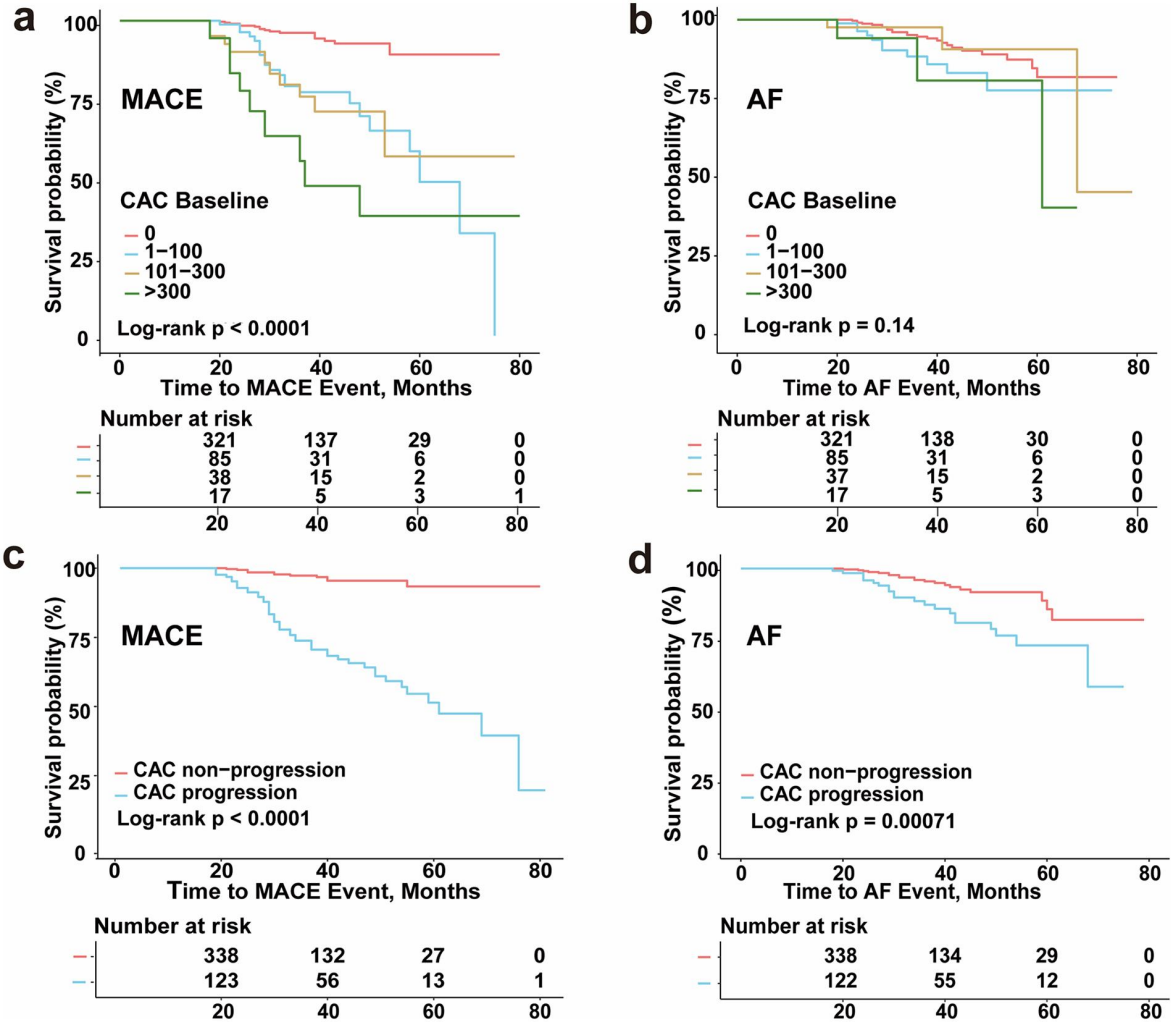
Associations of baseline CAC and CAC progression with clinical outcomes. Kaplan–Meier curves for major adverse cardiovascular events (MACE) and atrial fibrillation (AF) stratified by baseline CAC categories **(a,b)** and CAC progression status **(c,d)**.

Kaplan–Meier analyses demonstrated distinct associations between CAC and the two clinical endpoints. For MACE, both higher baseline CAC scores (Figure 3a) and the presence of CAC progression (Figure 3c) were associated with significantly worse event-free survival (log-rank *P* < 0.0001 for both). In contrast, no significant difference in AF-free survival was observed across baseline CAC severity categories (log-rank *p* = 0.14; Figure 3b). However, CAC progression was significantly associated with an increased incidence of AF (log-rank *P* = 0.00071; Figure 3d).

In univariable Cox regression analyses, age (HR 1.05, 95% CI 1.03–1.07; *P* < 0.001), hypertension (HR 2.50, 95% CI 1.53–4.21; *P* < 0.001), diabetes (HR 2.85, 95% CI 1.44–5.67; *P* = 0.003), and drinking (HR 2.10, 95% CI 1.18–3.73; *P* = 0.012) were significantly associated with MACE (Supplementary Table S7). In multivariable Cox regression models adjusting for age, hypertension, diabetes (Table 2), baseline CAC score, annualized CAC progression rate, and CAC progression group remained independently associated with MACE. In contrast, absolute change in CAC was not associated with MACE occurrence. Notably, the CAC progression group was associated with a markedly increased risk of MACE (HR 7.41; *P* < 0.001). Further analyses stratified by CAC progression groups (Table 3) revealed a graded risk pattern, with the highest risk observed in individuals with relative CAC progression (baseline CAC >100; HR 10.31), followed by those with absolute progression (baseline CAC1-100; HR 8.14) and those with incident CAC (baseline CAC = 0; HR 5.22; all *P* < 0.001).

**Table 2:** Adjusted HRs for CAC baseline and progression for MACE.

Variable	MACE	
	HR (95% CI)	*p* value
Adjusted for age, hypertension, diabetes		
CAC baseline	1.002 (1.001–1.003)	**0**.**003**
CAC baseline severity		
0	Ref	-
1–100	3.202 (1.569–6.538)	**0**.**001**
101–300	3.853 (1.656–8.962)	**0**.**002**
>300	5.669 (2.171–14.80)	**<0**.**001**
CAC absolute change	1.000 (0.999–1.001)	0.934
CAC progression per year	1.004 (1.002–1.007)	**<0**.**001**
CAC progression group	7.412 (3.715–14.79)	**<0**.**001**

Significant *P*-values are printed in bold. Abbreviations as in Table 1.

**Table 3:** Adjusted HRs for CAC progression groups for MACE.

Group	MACE	
	HR (95% CI)	*p* value
Non progression	Ref	–
Progression groups*		
CAC Incident (Baseline CAC = 0)	5.22 (2.155–12.620)	**<0**.**001**
Absolute CAC Progression (0< Baseline CAC < 100)	8.14 (3.682–17.986)	**<0**.**001**
Relative CAC Progression (Baseline CAC > 100)	10.31 (4.420–24.026)	**<0**.**001**

Significant *P*-values are printed in bold; Detailed definitions of progression groups provided in methods.

### 3.3 The prediction accuracy and risk reclassification of CAC progression

**Figure 4:**
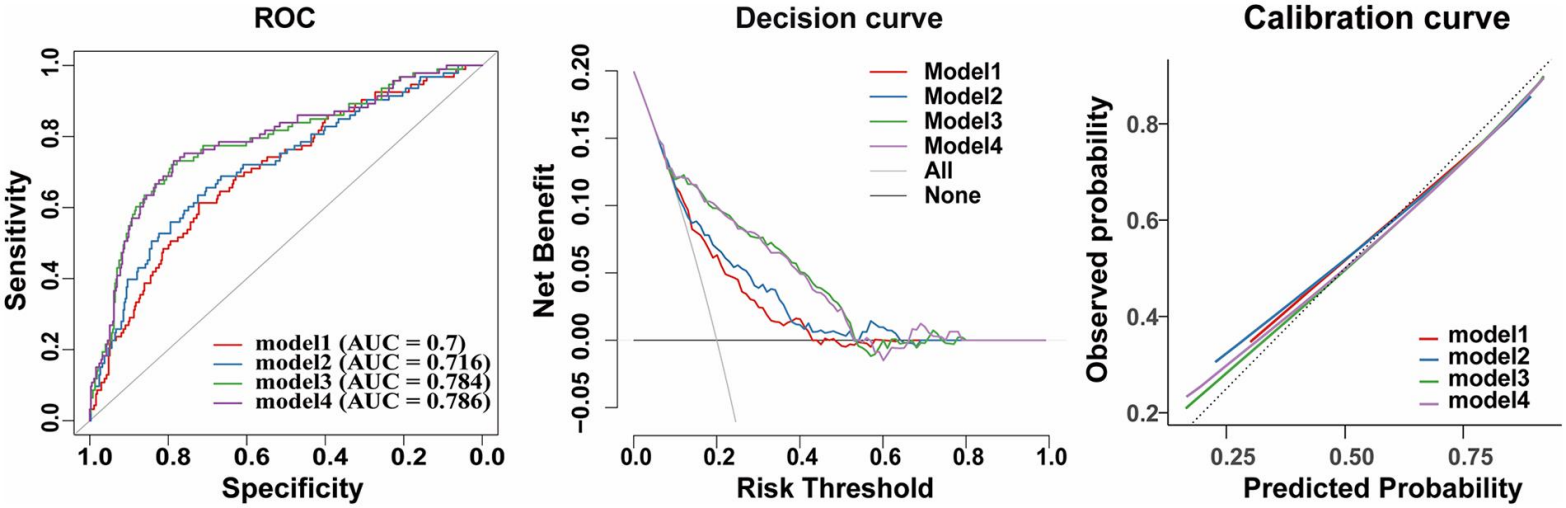
Performance of four prediction models for major adverse cardiovascular events. Receiver operating characteristic curves, calibration plots, and decision curve analyses comparing Models 1–4. Detailed definitions of Models 1–4 are provided in Table 3.

The prediction accuracy and risk reclassification of each model are shown in Table 4. The C-index and AIC of Model 1, which included age, hypertension and diabetes were 0.67 (95% CI 0.66–0.77) and 986.14, respectively. In constructing the multivariate Cox regression model for MACE, two key CAC parameters were selected based on clinical relevance and model parsimony: baseline CAC score and CAC progression group (as defined in the Methods). The addition of baseline CAC to Model 1 (Model 2) resulted in a statistically significant but modest improvement in the C-index [0.68 (95% CI 0.68–0.78), *P* = 0.021 vs. Model 1], with a non-significant NRI of 0.04 (95% CI −0.07–0.15). Incorporating CAC progression group to Model 1 (Model 3) substantially improved the model performance, yielding a significantly higher C-index [0.73 (95% CI 0.72–0.82), *P* < 0.001 vs. Model 1] and a significant NRI of 0.22 (95% CI 0.08–0.34). When both baseline CAC and CAC progression group were included (Model 4), the C-index [0.72 (95% CI 0.72–0.82)] was significantly improved compared to Model 2 (*P* < 0.001) with an NRI of 0.17 (95% CI 0.06–0.27). However, Model 4 did not offer a significant improvement over Model 3, as indicated by a non-significant NRI of −0.01 (95% CI −0.04–0.01). The lowest AIC value was observed in Model 3 (949.61). Figure 4 compares model performance using time-dependent ROC, decision curve, and calibration analyses. The traditional risk factor model (Model 1) showed modest discrimination for MACE (AUC = 0.70). Adding baseline CAC slightly improved performance (Model 2, AUC = 0.716), whereas inclusion of CAC progression markedly increased discrimination (Model 3, AUC = 0.784). Adding baseline CAC to CAC progression (Model 4) did not further improve model performance. Decision curve and calibration analyses supported the superior clinical utility and calibration of models incorporating CAC progression.

**Table 4:** Incremental prognostic utility for MACE in different models.

Model	Included variables	C-index (95% CI)	*p* value	NRI (95% CI)	AIC
1^a^	Age, hypertension, diabetes	0.67 (0.66–0.77)	–	–	986.14
2	Model 1 + CAC baseline	0.68 (0.68–0.78)	vs. Model 1 ***P*** **=** **0.021**	vs. Model 1 0.04 (−0.07–0.15)	982.42
3	Model 1 + CAC progression group	0.73 (0.72–0.82)	vs. Model 1 ***P*** **<** **0.001**	vs. Model 1 0.22 (0.08–0.34)	949.61
4	Model 2 + CAC progression group	0.72 (0.72–0.82)	vs. Model 2 ***P*** **<** **0.001**	vs. Model 2 0.17 (0.06–0.27) vs. Model 3 −0.01 (−0.04–0.01)	954.32

Significant *P*-values are printed in bold; AIC, Akaike information criterion; CI, confidence interval; NRI, net reclassification index.

a Model 1 included age, hypertension, and diabetes, selected based on univariable analyses (*P* < 0.001).

## 4 Discussion

To our knowledge, this is the first longitudinal study to evaluate the prognostic value of CAC progression for cardiovascular risk in patients with IBD, and the largest study to date assessing the utility of CAC in this population. Using opportunistic non-gated chest CTs obtained during routine IBD care, we assessed cardiovascular risk without additional radiation or cost, in a cohort likely more representative of at-risk IBD patients than cardiac referral populations. Our main findings are: (1) CAC progression independently predicts MACE, providing incremental prognostic value beyond traditional risk factors and baseline CAC; (2) incident CAC in patients with baseline CAC = 0 identifies a higher-risk subgroup who may benefit from intensified management; and (3) CAC progression is preliminarily associated with incident atrial fibrillation, a relationship not observed with baseline CAC.

Previous studies have described an “ASCVD paradox” in IBD, with increased ASCVD risk despite a low burden of traditional cardiovascular risk factors (4, 21, 22). A cross-sectional studies found IBD patients have a distinct cardiovascular risk profile characterized by a lower prevalence of overweight and hypercholesterolaemia but a higher prevalence of hypertension, abdominal obesity, and hypertriglyceridaemia compared to age/gender-matched controls (23). A study of 60,155 U.S. adults without ASCVD also suggested early screening for hypertension and diabetes and reinforced lifestyle interventions, particularly in younger patients with IBD (21). In line with prior observations, our findings suggest MACE mainly concentrated among patients with hypertension and diabetes, whereas BMI, smoking status, and lipid profiles were similar between groups. Several investigations further highlight the need to pay particular attention to cardiovascular risk management in younger IBD patients especially women (1, 24). In our study, no significant gender-related difference was observed, but this result should be interpreted cautiously due to the limited sample size and event number.

CAC assessment has emerged as a widely available, cost- and time-efficient tool for improving CVD risk stratification and guiding primary prevention strategies in asymptomatic individuals (6). Its application has subsequently been extended to other clinical settings, including patients with IBD (15, 16). In a retrospective study of 369 patients with IBD, Naami et al. demonstrated that CAC may better capture the excess cardiovascular risk driven by chronic inflammation in IBD than traditional risk calculators, and may more directly inform clinical decision-making (15). In our cohort, the association between CAC and MACE was contingent on both the baseline burden of CAC and its progression over time. Notably, CAC progression remained a robust and sensitive prognostic marker for MACE even after adjustment for traditional cardiovascular risk factors in multivariable models. Further analyses revealed a graded relationship between distinct CAC progression groups and incident MACE, independent of conventional risk factors. From a clinical perspective, the “incident CAC” pattern underscores the prognostic relevance of CAC progression among individuals with no detectable CAC at baseline. Although such individuals are generally classified as low risk based on a single CAC assessment, the development of incident CAC may warrant reclassification into a higher-risk category, reflecting a substantially increased likelihood of subsequent MACE. Importantly, absolute changes in CAC scores between scans were not significantly associated with MACE, suggesting that binary or pattern-based definitions of CAC progression may better capture clinically meaningful atherosclerotic activity than continuous absolute changes, particularly in the context of IBD-related vascular inflammation (13, 25). Moreover, incorporating CAC progression into conventional risk prediction models resulted in significant improvements in both discrimination and risk reclassification. In contrast, baseline CAC added only modest incremental value. Collectively, these findings reinforce the concept that in chronic inflammatory conditions, dynamic imaging markers may more accurately reflect the evolving atherosclerotic process and its clinical consequences than static measurements obtained at a single time point (26).

A novel and intriguing finding of our study is the specific association between CAC progression, but not baseline CAC, and incident AF, which align with the prior longitudinal evidence from the MESA cohort (27). Previous research reported an increased risk of AF in IBD patients in comparison to the general population (2, 28). This finding supports the concept that dynamic atherosclerotic progression reflects an active inflammatory and pro-arrhythmic milieu, which may promote atrial structural and electrical remodeling and thereby increase AF susceptibility, especially in the context of IBD-related systemic inflammation. In the cardiac setting, AF is well described as a chronic rather than acute condition, which is in keeping with the premise of IBD as persistent systemic inflammatory state and warrants further investigation in future studies.

We initially hypothesized that disease severity might help explain the progression of CAC, as sustained inflammatory states are theoretically expected to accelerate systemic atherosclerosis and subsequent calcium deposition. Contrary to this expectation, we did not find a significant association between IBD-related characteristic (such as disease severity, duration, or CRP levels) and the presence of CAC progression. Prior studies have shown that associations between baseline CRP levels and vascular calcification are inconsistent, underscoring the limitations of single-time-point inflammatory markers in reflecting chronic inflammatory exposure (29, 30). Moreover, clinical classifications of disease severity may not fully reflect the specific molecular and subclinical inflammatory pathways that drive atherosclerosis progression, which involve complex innate and adaptive immune signaling beyond traditional clinical metrics (31). Therefore, rather than refuting the role of inflammation, our findings suggest that CAC progression may represent a more integrative downstream marker of the cumulative vascular consequences of IBD-related inflammation than traditional measures of disease activity.

This study has several limitations. First, the study was conducted in two tertiary hospitals in China, where patients may present with more severe disease, and its characteristics may not be fully generalizable to all IBD populations globally. Second, cause-and-effect relationships cannot be inferred from this retrospective observational study. Third, the interscan interval for CAC assessment varied among patients, and CAC progression is not linear, the association between CAC progression and MACE may differ across follow-up durations. Fourth, the total number of MACE events, while significant, limits the statistical power for more detailed subgroup analyses. Finally, our data on IBD-related factors, such as cumulative drug exposure (e.g., corticosteroids, biologics) and long-term disease activity, were limited, preventing a more granular analysis of their impact on CAC progression. To this end, it is important that future longitudinal studies assess the association between traditional and IBD-related risk factors with CAC and its progression (both disease characteristics and medication use) and their use in risk stratification.

In conclusion, CAC progression independently predicts MACE and is associated with incident AF in patients with IBD, providing prognostic value beyond baseline CAC and traditional risk factors. Opportunistic assessment of CAC on routine chest CT may help identify high-risk patients and guide earlier preventive strategies, highlighting the importance of dynamic atherosclerosis evaluation in chronic inflammatory conditions.

## Data Availability

The original contributions presented in the study are included in the article/Supplementary Material, further inquiries can be directed to the corresponding author/s.
