# Supplementary material for: Opportunistic coronary calcium progression on routine chest CT improves cardiovascular risk stratification in patients with inflammatory bowel disease: a multicenter study
Source: Front Cardiovasc Med. 2026 Apr 28;13:1804400. doi: 10.3389/fcvm.2026.1804400 (PMC13132753; doi:10.3389/fcvm.2026.1804400)
Supplement: Supplementary file 1 [file Datasheet1.pdf]

Supplement materials

Contents

1. Formula for Calculating the Annual Rate of CAC Progression
2. **Supplement Table 1** Baseline Characteristics of Patients Stratified by CAC
3. **Supplement Table 2** Comparison of Clinical Characteristics and Outcomes between Ulcerative Colitis and Crohn's Disease
4. **Supplement Table 3** Comparison of Clinical Characteristics and Outcomes Stratified by IBD Disease Activity at Baseline
5. **Supplement Table 4** Comparison of CAC Progression and Clinical Outcomes Stratified by Biologics Use Patterns
6. **Supplement Figure 1** Major Adverse Cardiovascular Events across Age Groups
7. **Supplement Table 5** Individual Components of Major Adverse Cardiovascular Events
8. **Supplement Table 6** Characteristics of Patients Stratified by Major Adverse Cardiovascular Events
9. **Supplement Table 7** Univariable Cox Regression Analysis to Predict Major Adverse Cardiovascular Events

Formula for Calculating the Annual Rate of CAC Progression

1.For participants with 0 < baseline CAC < 100, annualized absolute progression was calculated as:

$$\text{Annualized CAC change} = \frac{\text{Follow-up CAC} - \text{Baseline CAC}}{\text{Time interval in years}}$$

2.For participants with baseline CAC ≥100, the annualized relative progression was calculated as:

$$\text{Annualized relative progression} = \frac{\text{Follow-up CAC} - \text{Baseline CAC}}{\text{Baseline CAC} \times \text{Time interval in years}} \times 100\%$$

Supplement Table 1. Baseline Characteristics of Patients Stratified by CAC

Characteristics	Total (N=467)	Baseline CAC=0 (n=322)	Baseline CAC>0 (n=145)	*P* value
Age, years	54.1 ± 13.1	50.0 ± 12.0	63.0 ± 10.7	**<0.001**
Male, %	262 (56.1)	164 (50.9)	98 (67.6)	**<0.001**
Moderate to severe IBD, %	300 (64.2%)	209 (64.9）	91 (62.8)	0.654
Disease type, %				0.477
UC	338 (72.4%)	228 (70.8%)	110 (75.9%)	
CD	103 (22.1%)	76 (23.6%)	27 (18.6%)	
IBD-U	26 (5.6%)	18 (5.6%)	8 (5.5%)	
Age at diagnosis				**<0.001**
18 to <40	61 (32.1%)	53 (42.7%)	8 (12.1%)	
40 to <60	82 (43.2%)	51 (41.1%)	31 (47.0%)	
≥60	47 (24.7%）	20 (16.1%)	27 (40.9%)	
Disease duration, years	4 (1, 10)	4 (1, 10)	4 (1, 12)	0.800
BMI, kg/m^2	22 (20, 25)	22 (20, 24)	23 (21, 25)	0.029
SBP, mmHg	121 (111, 130)	120 (110, 128)	125 (115, 136)	**<0.001**
Smoking status, %				**0.006**
Never smoker	354 (75.8)	256 (79.5)	98 (67.6)	
Former smoker	63 (13.5)	33 (10.3)	30 (20.7)	
Current smoker	50 (10.7)	33 (10.3)	17 (11.7)	
Drinking, %	72 (15.4)	38 (11.8)	34 (23.5)	**0.001**
Hypertension, %	110 (23.6)	54 (16.8)	56 (38.6)	**<0.001**
Diabetes, %	40 (8.6)	16 (4.97)	24 (16.6)	**<0.001**
Antihypertensives, %	92 (19.7)	46 (14.3)	46 (31.7)	**<0.001**
Sleep problems, %	108 (23.1)	76 (23.6)	32 (22.1)	0.716
Aspirin, %	12 (2.6)	3 (0.9)	9 (6.2)	**0.003**
Corticosteroids, %	148 (31.7)	107 (33.2)	41 (28.3)	0.287
Aminosalicylates, %	407 (87.2)	288 (89.4)	119 (82.1)	**0.028**
Biologicals, %	234 (50.1)	180 (55.9)	54 (37.2)	**<0.001**
CRP, mg/L	3.5 (1.1, 16.6)	3.1 (1.1, 14.0)	4.8 (1.3, 24.6)	**0.015**
Dyslipidemia, %	132 (28.3)	82 (25.5)	50 (34.5)	**0.045**
TC (mmol/L)	4.0 (3.4, 4.9)	4.1 (3.4, 4.8)	3.9 (3.3, 5.1)	0.764
LDL (mmol/L)	2.04 (1.3, 2.7)	2.0 (1.3, 2.7)	2.0 (1.3, 2.8)	0.828
HDL (mmol/L)	1.0 (0.9, 1.4)	1.1 (0.9, 1.4)	1.0 (0.8, 1.4)	0.885
Trigylcerides (mmol/L)	1.5 (1.0,2.6)	1.6 (1.0, 2.5)	1.5 (1.0,2.6)	0.826
Fasting glucose	4.7 (4.3,5.2)	4.7 (4.2, 5.1)	4.9 (4.4,5.4)	**0.003**
CAC progression, %	129 (27.6)	40 (12.4)	89 (61.4)	**<0.001**

Significant P-values are printed in bold. CAC: coronary artery calcium; BMI: body mass index; SBP: systolic blood pressure; DBP: diastolic blood pressure; TC: total cholesterol; LDL-C: low-density lipoprotein-cholesterol; CRP:C-reactive protein.

Supplement Table 2. Comparison of Clinical Characteristics and Outcomes between Ulcerative Colitis and Crohn's Disease

Characteristics	UC (n=338)	CD (n=103)	*P* value
Age, years	54.8 ± 13.1	52.0 ± 13.0	0.056
Male, %	195 (57.7)	56 (54.3)	0.551
BMI, kg/m^2	22.7 ± 3.4	22.1 ± 3.5	0.117
Hypertension, %	80 (23.7)	27 (26.2)	0.598
Diabetes, %	29 (8.6)	9 (8.7)	0.960
Hyperlipidemia, %	86 (25.4)	35 (34.0)	0.089
TC (mmol/L)	4.1 (3.4, 4.8)	3.8 (3.3, 4.7)	0.263
LDL (mmol/L)	1.6 (1.1, 2.54)	1.9 (1.3, 2.7)	**＜0.001**
CRP, mg/L	4.1 (1.1, 17.5)	2.9 (0.9, 13.0)	0.130
Baseline CAC category			0.224
Baseline CAC=0, %	228 (67.46)	76 (73.79)	
Baseline CAC>0, %	110 (32.5)	27 (26.2)	
CAC progression, %	92 (27.2)	34 (33.0)	0.255
MACE, %	45 (13.3)	11 (10.7)	0.482
AF, %	29 (8.6)	10 (9.7)	0.724

Supplement Table 3. Comparison of Clinical Characteristics and Outcomes Stratified by IBD Disease Activity at Baseline

Characteristics	Active disease (n=369)	Remission (n=98)	*P* value
Age, years	54.2 ± 12.9	53.7 ± 14.0	0.758
Male, %	198 (53.7)	64 (65.3)	**0.039**
BMI, kg/m^2	22.5 ± 3.4	22.8 ± 3.4	0.427
Hypertension, %	85 (23.0)	25 (25.5)	0.608
Diabetes, %	32 (8.7)	8 (8.2)	0.873
Hyperlipidemia, %	105 (28.5)	27 (27.6)	0.86
TC (mmol/L)	4.00 (3.4, 4.9)	4.1 (3.4, 4.7)	0.955
LDL (mmol/L)	2.0 (1.3, 2.7)	2.1 (1.4, 2.9)	0.251
CRP, mg/L	4.5 (1.2, 19.1)	2.3 (1.0, 6.3)	**0.002**
Baseline CAC category			0.575
Baseline CAC=0, %	277 (75.07)	61 (62.24)	
Baseline CAC>0, %	92 (24.93)	37 (37.76)	
CAC progression, %	92 (24.93)	37 (37.76)	**0.012**
MACE, %	47 (12.74)	12 (12.24)	0.896
AF, %	31 (8.40)	10 (10.20)	0.575

Supplement Table 4. Comparison of CAC Progression and Clinical Outcomes Stratified by Biologics Use Patterns

Characteristics	No biologics during study (n=210)	Biologics used at baseline or follow-up (n=257)	*P* value
CAC progression, %	53 (25.2)	76 (29.6)	0.297
MACE, %	34 (16.2)	25 (9.7)	**0.037**
AF, %	18 (8.6)	23 (9.0)	0.886

Supplement Figure 1. Major Adverse Cardiovascular Events across Age Groups

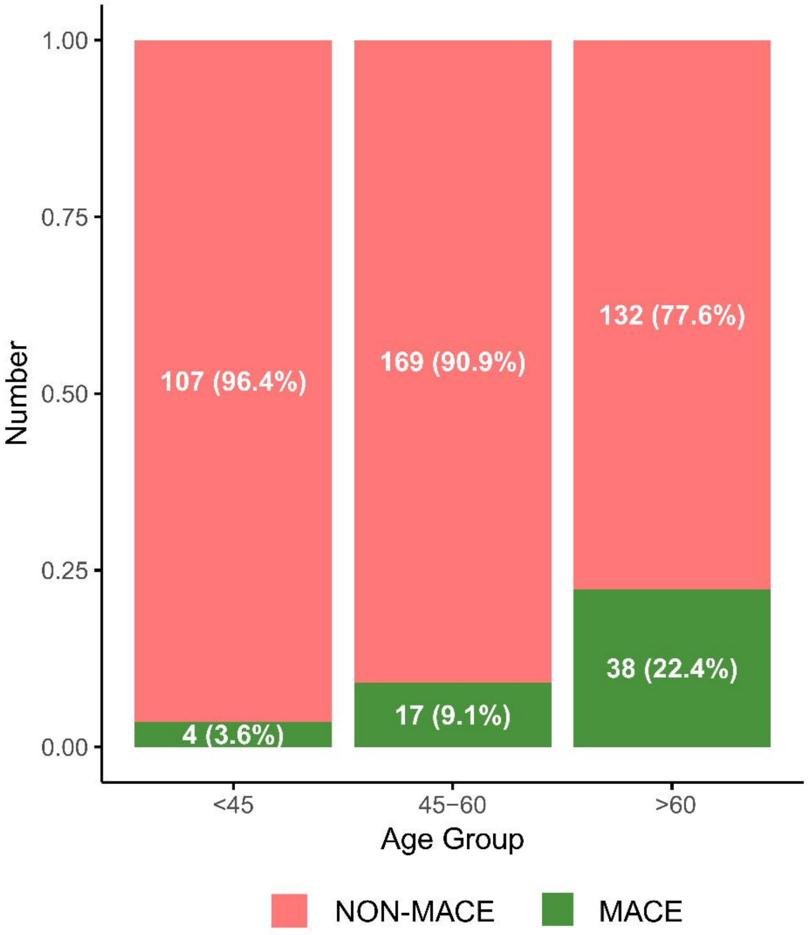

Bar chart showing the distribution of major adverse cardiovascular events (MACE) across different age groups during follow-up.

Supplement Table 5. Components of Major Adverse Cardiovascular Events

Outcomes	Number of events (rate)
Myocardial infarction	2(3.4%)
Unstable angina	21(35.6%)
Ischemic stroke	15(25.4%)
Transient ischemic attack	5(8.5%)
Peripheral arterial ischemia	2(3.4%)
Coronary revascularization (PCI/CABG)	10(16.9%)
All-cause mortality	4(6.8%)

Supplement Table 6. Characteristics of Patients Stratified by MACE

Characteristics	Total (N=467)	NONE MACE (n=408)	MACE (n=59)	*P* value
Age, years	54.1 ± 13.1	53.0 ± 13.0	61.5 ± 11.2	**<0.001**
Male, %	262 (56.1)	222 (54.4)	40 (67.8)	0.053
Moderate to severe IBD, %	300 (64.2)	266 (65.2)	34 (57.6)	0.421
Disease type, %				0.769
UC	338 (72.4)	293 (71.8)	45 (76.3)	
CD	103 (22.1)	92 (22.6)	11 (18.6)	
IBD-U	26 (5.6)	23 (5.6)	3 (5.1)	
Disease duration, years	7.0 ± 7.6	6.8 ± 7.4	8.5 ± 9.1	0.102
BMI, kg/m^2	22.5 ± 3.4	22.4 ± 3.4	23.2 ± 3.6	0.109
Former smoker	63 (13.5)	51 (12.5)	12 (20.3)	0.099
Current smoker	50 (10.7)	44 (10.8)	6 (10.2)	0.886
Drinking%	72 (15.4)	56 (13.7)	16 (27.1)	**0.008**
Hypertension, %	110 (23.6)	84 (20.6)	26 (44.1)	**<0.001**
Diabetes, %	40 (8.6)	30 (7.4)	10 (17.0)	**0.014**
Hyperlipidemia, %	132 (28.3)	109 (26.7)	23 (39.0)	0.050
TC (mmol/L)	4.0 (3.4, 4.9)	4.1 (3.4, 4.8)	3.7 (3.2, 5.1)	0.299
LDL (mmol/L)	2.0 (1.3, 2.7)	2.1(1.3, 2.7)	1.9 (1.3, 2.7)	0.722
Aspirin, %	12 (2.6)	5 (1.2)	7 (11.9)	**<0.001**
Corticosteroids, %	148 (31.7)	128 (31.4)	20 (33.9)	0.697
Aminosalicylates, %	407 (87.2)	358 (87.8)	49 (83.1)	0.314
Biologicals, %	234 (50.1)	210 (51.5)	24 (40.7)	0.121
CRP, mg/L	3.5 (1.1, 16.6)	3.5 (1.1, 16.9)	3.9 (1.1, 15.0)	0.980
Fasting glucose	4.7 (4.3, 5.2)	4.7 (4.3, 5.2)	4.9 (4.4, 5.6)	**0.032**
CAC Change	0 (0, 4)	0 (0, 1.9)	35.9 (4, 120.5)	**＜0.001**
CAC progression per year	0 (0, 2.7)	0 (0, 0.9)	22.0 (3.8, 74.8)	＜0.001
CAC progression group, %	129 (27.6)	82 (20.1)	47 (79.7)	＜0.001
CAC inter-scan time, months	19 (13, 25)	20 (13, 25)	14 (12, 24)	0.012

Supplement Table 7. Univariable Cox regression analysis to predict MACE

	Unadjusted	
	HR (95% CI)	*P* value
Age	1.05 (1.02-1.07)	**<0.001**
Gender	0.66 (0.38-1.1)	0.13
Diagnosis Age	1.02 (1.00-1.23)	**<0.001**
Hypertension	2.50 (1.53-4.21)	**<0.001**
Diabetes	2.85 (1.44-5.67)	**0.0028**
Hyperlipidemia	1.72 (1.01-2.92)	**0.049**
BMI	1.00 (0.98-1.14)	0.16
Smoking	1.44 (0.82-2.50)	0.2
Drinking	2.10 (1.18-3.73)	**0.012**
TC	0.95 (0.74-1.21)	0.67
LDL	1.02 (0.77-1.34)	0.9
CAC Baseline	1.00 (1.00-1.00)	**<0.001**
CAC absolute change	1.00 (1.00-1.00)	0.064
CAC Progression per year	1.08 (1.05-1.09)	**<0.001**
CAC Progression group	10.21 (5.4-19.01)	**<0.001**
